# Biocatalytic Synthesis of Diethyl Succinate in Hydrophobic Trioctylphosphine Oxide (TOPO)-Based Reactive DES

**DOI:** 10.3390/molecules31193402

**Published:** 2026-09-24

**Authors:** Susana Nieto, Francisco J. Ruiz, Juan Martinez-Garcia, Rocio Villa, Jairton Dupont, Pedro Lozano

**Affiliations:** Departamento de Bioquímica y Biología Molecular “B” e Inmunología, Facultad de Química, Universidad de Murcia, 30100 Murcia, Spain

**Keywords:** TOPO, reactive DES, diethyl succinate, sustainable chemistry, eutectic mixture

## Abstract

Diethyl succinate (DEtS) synthesis by lipase-catalyzed direct esterification was successfully carried out in succinic acid (SA):trioctylphosphine oxide (TOPO) mixtures acting as reactive deep eutectic solvents (rDES). By this strategy, the succinic acid concentration in this non-aqueous eutectic mixture reaches up to 1.92 M at room temperature, enabling a suitable reaction system for the biocatalytic double esterification of SA with ethanol (EtOH) to yield DEtS. The best results were obtained when using an SA:TOPO equimolar ratio and a stepwise addition of EtOHinto the reaction system, leading to 92% SA conversion and 60% selectivity for the DEtS product synthesis after 20 h at 60 °C. For the subsequent pure product separation, the direct addition of dimethyl sulfoxide into the reaction system provokes TOPO crystallization, which is easily removed by filter centrifugation at room temperature. This novel approach, TOPO-based rDES, has been demonstrated as a green and low-waste strategy for biocatalytic diester synthesis under mild conditions, validated by green metrics.

## 1. Introduction

Conventional models of consumption and chemical transformations have led to the current depletion of fossil resources. This situation is driving the search for new biobased feedstocks obtained through more environmentally friendly transformations guided by the philosophies of Green Chemistry [1] and Circular Chemistry [2], setting the focus on minimizing waste by reducing its generation and promoting its reintroduction in the production chain, respectively.

Diethyl succinate (DEtS) has arisen as a greener alternative to fossil-derived volatile solvents due to its lower toxicity and volatility and higher biodegradability. Currently, the market for DEtS is estimated to reach $148.6 million in 2025, with a Compound Annual Growth Rate (CAGR) of 8.1% for the period 2026–2034 [3]. This growth has been boosted by the development of new sources for obtaining succinic acid (SA). Different microbial strains capable of synthesizing SA have been identified (e.g., *Anaerobiopirillum succiniciproducens*, *Actinobacillus succinogenes*, *Mannheimia succiniproducens*) or biotechnologically designed (i.e., *E. coli*) to improve SA production and cost-effectiveness [4]. The bio-based production of SA through biomass fermentation has efficiently improved the production rate, while contributing to the valorization of waste derived from other sectors (e.g., lignin, cellulose, glycerol, CO_2_) [5]. In this context, it was reported that 61.5% of SA used as DEtS in 2025 had a bio-based origin [1].

Furthermore, DEtS is a valuable building block in the synthesis of biodegradable polymers, like polybutylene succinate (PBS) [6], obtained by polycondensation with 1,4-butanediol (BDO) through transesterification reactions. This polymer is gaining relevance because of its biodegradability, similar physicochemical properties to polyolefins, and wide applicability [7]. The greater suitability of DEtS than SA to obtain PBS relies on its higher miscibility with BDO at low temperatures, which is more suitable for biocatalytic synthesis and avoids uncontrolled secondary reactions. It should be noted that high SA concentrations may also acidify the medium, which may affect the conformational stability of the biocatalysts, making the use of SA derivatives preferable. In this regard, the advantage of using DEtS lies in the high volatility of the EtOH byproduct, whose continuous removal from the reaction medium shifts the equilibrium towards PBS synthesis. However, the synthesis of DEtS is hampered by the low SA solubility in non-aqueous solvents, the immiscibility with EtOH, and thermodynamic limitations. [8] Different strategies have been developed to overcome these drawbacks, such as the acidification of SA and further esterification with EtOH catalyzed by H_2_SO_4_ [9]. López-Garzón et al. [10] further optimize this protocol, skipping the need for SA acidification by using an anion exchange column that acts at the same time as a catalyst for SA esterification with chloroethane, achieving up to 71% DEtS in 1 h at 100 °C. Orjuela et al. [8] also reported a complex reactive distillation system for DEtS synthesis catalyzed by Amberlyst 70 using an excess of EtOH, achieving up to 98% yield.

This scenario underscores the need to improve the sustainability of DEtS synthesis by replacing organic and metal catalysts, whose deposition on the products can restrict applications in sectors such as medicine [11], with a biodegradable catalyst. The use of biocatalysis enhances energy efficiency and prevents EtOH dehydration to diethyl ether, improving mass efficiency [8].

Additionally, SA solubilization remains a major challenge. Here, reported strategies for bio-based SA solubilization and extraction from fermentation broth serve as a reliable starting point. Ionic liquids (ILs) offer a promising alternative to costly and low-efficiency conventional methodologies (e.g., precipitation, distillation, electrodialysis, crystallization) for SA extraction because of their efficient solubilization capacity [12]. As a representative example, the aqueous two-phase system using the IL [C_8_mim][Br] and an aqueous solution of 20% (NH_4_)_2_SO_4_ enabled 85.5% SA extraction. However, the downstream process, based on several steps of precipitation with sodium hydroxide and crystallization of sodium succinate, remained poorly sustainable [13]. Phosphonium ILs have also demonstrated their suitability for the extraction of diluted short organic acids from aqueous phases [14,15]. COSMO-RS analyses performed by Cabezas et al. [16] demonstrated the capability of the IL trihexyl tetradecyl phosphonium bis(2,4,4-trimethylpentyl) phosphinate, [P_6,6,6,14_][PHOS] (Scheme 1), to establish hydrogen bonding with SA, replacing the hydrogen interaction between SA and water. Consequently, efficient and selective SA extraction was achieved using this IL from a simulated fermentation broth also containing glycerol. Going beyond, these authors immobilized this IL in a membrane between two layers of cellulose acetate to develop an efficient system for SA perstraction from fermentation broths, reaching 43.1% efficiency in SA extraction and a selectivity SA/glycerol of 55 [17].

The technology of deep eutectic solvents also offers a valuable platform to address the solubilization and esterification of SA. By definition, eutectic mixtures are liquid homogeneous media obtained after a network of hydrogen bonding is formed between their solid counterparts [18], sharing many characteristics with ILs (i.e., cooperative interactions, dynamic association equilibria, local structural organization) [19]. These mixtures are obtained from species acting as hydrogen bond donors (HBDs) and hydrogen bond acceptors (HBAs) that establish a network of hydrogen bonding, which leads to a significant depression of the melting temperature of the mixture, resulting in a homogeneous liquid phase [20]. Thus, the technology of eutectic mixtures offers a valuable opportunity to address the solubilization and biocatalytic transformation of substrates under mild conditions. Moreover, to maximize sustainability and material efficiency in these systems, reactive DESs (rDESs) can be designed using a substrate as part of the eutectic mixture, or as fully solventless “2-in-1” systems composed exclusively of the reaction substrates. This last approach has enabled the solventless esterification of free fatty acids with panthenol and the lipophilization of aromatic compounds [21,22,23], enhancing materials efficiency and sustainability. Notably, operating under solvent-free conditions while achieving high reaction yields allows for direct product utilization, thereby circumventing the need for costly downstream separation and purification steps.

TOPO (tri-n-octyl phosphine oxide) is a phosphorus-bonded, oxygen-bearing compound, resembling the IL [P_6,6,6,14_][PHOS] (see Scheme 1), with reported capability to form eutectic mixtures with dicarboxylic acids used for metal extraction [24] or the extraction of short organic acids from aqueous phases [25]. The extraction capability of TOPO is attributed to its phosphoryl group, whose strong Lewis base character enables the formation of hydrogen-bonding interactions with the organic acid, thereby facilitating its extraction [26].

This work explores the use of TOPO to directly form a rDES with SA for the further biocatalytic synthesis of DEtS after the addition of EtOH as co-substrate (Figure 1). It should be highlighted that, to date, there is no mention in the literature about the suitability of TOPO to support biocatalysis, with only the use of TOPO as a complexing agent in a two-phase system with ILs for synthesizing caffeic acid phenethyl ester (CAPE) being reported [27]. Thus, this work addresses the identification of suitable conditions for obtaining the eutectic mixture and further biocatalytic esterification to surpass the thermodynamic equilibrium and improve the biocatalytic yield under mild reaction conditions. Additionally, a clean approach for the obtention of pure products and TOPO recovery is addressed, as well as the quantitative validation of this strategy’s sustainability by means of green metrics analysis.

## 2. Results

### 2.1. Biocatalytic Synthesis of Diethyl Succinate in TOPO-Based rDES

SA is characterized by poor solubility in most organic solvents. To overcome this hindrance, the feasibility of obtaining eutectic mixtures with TOPO was analyzed. Using a fixed amount of 5 mmol SA, mixtures with different molar ratios of TOPO:SA (2:1; 1.5:1; 1:1 and 1:2 mol/mol) were prepared (Figure 2A) and vigorously mixed by magnetic stirring at 70 °C for 1 h (Figure 2B). After cooling to room temperature, a visual inspection identified the mixture that remained as a homogeneous liquid at room temperature, indicating that the eutectic point had been reached. Only the 2:1 TOPO:SA molar ratio completely fulfilled that requirement. While 1.5:1 and 1:1 TOPO:SA molar ratios also provided homogeneous systems, a clearly visible fraction of solid SA indicated the formation of partial eutectic mixtures. Only the 1:2 TOPO:SA molar ratio remained a heterogeneous system with the highest amount of precipitated SA. However, neither vial contained solid TOPO, suggesting its complete solubilization upon formation of the eutectic mixture.

ATR-FTIR analyses of TOPO and SA standards and the liquid phase of each vial further corroborated the formation of eutectic mixtures (Figure S1). The FTIR spectrum of the 2:1 TOPO:SA molar ratio, as a model of a full eutectic mixture, confirmed the hydrogen bonding interactions between both counterparts, evidenced by the shift of the carbonyl group in SA from 1682 cm^−1^ to 1711 cm^−1^, and the redshift of the P=O stretching group from 1145 cm^−1^ to 1125 cm^−1^ (Figure S1A) [22]. The same shifts were detected in the liquid phase of the rest of the molar ratios, indicating the full interaction of TOPO with SA (Figure S1B). Therefore, the remaining solid SA may be caused by a deficit of TOPO in the mixture. Differential scanning calorimetry (DSC) analyses further provided the final confirmation. Mixtures of 2:1 and 1:1 TOPO:SA were selected as reaction models of full and partial eutectic mixtures to carry out this analysis (Figure S2). The linear spectrum of 2:1 TOPO:SA indicates that both components are liquid in the range of 40–220 °C, also confirming the eutectic mixture. For the case of the 1:1 TOPO:SA mixture, only a broad peak at 173.1 °C is observed, which may correspond to the remaining solid SA.

In this context, TOPO, as a phosphorus-bonded oxygen-bearing molecule, has proven capacity to interact with dicarboxylic acids, such as malic acid, enabling their separation from aqueous media when used in mixtures with ILs or organic solvents [28]. Furthermore, its capacity to form eutectic mixtures with long-chain organic acids (i.e., decanoic and dodecanoic acids) has been used for the extraction of dicarboxylic acids, including SA [25]. It should be noted that this approach of eutectic mixtures based on TOPO as a hydrogen bond acceptor (HBA) enables the easy solubilization of SA at 70 °C, a temperature much lower than its melting point (184–186 °C). However, full SA solubilization could only be achieved in the 2:1 TOPO:SA (mol/mol) mixture. While 1.5:1 or 1:1 molar ratios also enabled homogeneous liquid media, SA is not totally incorporated into the eutectic mixture. This restricted capacity of TOPO could be explained by the process stoichiometry, as TOPO possesses only a single P=O group acting as HBA, compared to the two carboxyl groups in SA acting as hydrogen bond donors (HBD).

Subsequently, all these mixtures were used for the biocatalytic synthesis of DEtS after the addition of EtOH as co-substrate (using a 4:1 EtOH:SA molar ratio), and N435 (33 mg/mmol SA) was incubated at 60 °C. Aliquots from the liquid phase were withdrawn at different incubation times to monitor the reactions by ^1^H-NMR analyses, quantifying the abundance of SA and the monoethyl succinate (MEtS) and DEtS products through the reaction using *tert*-butanol 100 mM as an internal standard (see Figure S3). SA conversion and reaction selectivity towards DEtS synthesis were determined according to Section 3.6.

Results shown in Table 1 demonstrate that the mixtures based on TOPO:SA effectively act as a rDES, providing the substrate SA for the biocatalytic direct esterification with EtOH to synthesize mono- and di-ester species. It should be pointed out that no uncontrolled side reactions were detected during the formation of the eutectic mixture or in a control reaction without enzyme, confirming the selective biocatalytic synthesis.

The apparent pH of the eutectic mixtures measured with a glass electrode revealed values between 4.2 and 4.4. Although the concept of pH, as defined for conventional aqueous solutions, cannot be directly applied to DESs, the apparent pH provides valuable information on the proton activity within the eutectic medium. This parameter influences the interactions between DES components and dissolved compounds and is also critical for maintaining enzyme stability and catalytic performance [29,30]. The addition of EtOH and the progress of the biocatalytic reaction resulted in a slight decrease in the apparent pH, suggesting a reorganization of the hydrogen-bonding network during esterification. These values are consistent with the optimal pH range reported for the N435-catalyzed esterification of succinic acid (pH 2–4) determined by Delhomme et al. [31]. In this context, it has been proposed that only the fully protonated form of SA (pKa_1_ = 4.21) acts as the preferred substrate, whereas the presence of a deprotonated carboxyl group may lead to electrostatic repulsion with the enzyme active site, thereby reducing catalytic efficiency [32,33].

The full rDES, based on a 2:1 TOPO:SA molar ratio, enabled up to 80% conversion in 20 h at 60 °C (entry 1). Unexpectedly, this performance improved gradually with the decrease of TOPO content in the mixture, achieving up to 86% conversion with the equimolar ratio (entry 3). Although the 1.5:1 and 1:1 TOPO:SA mixtures did not provide fully eutectic mixtures, they were observed to gradually evolve into completely solubilized homogeneous systems during the reaction (Figure 2C). These results suggest that the precipitated SA acts as a reservoir, enabling hydrogen-bond interactions with TOPO and maintaining the eutectic mixture as the solubilized SA is biocatalytically esterified to MEtS and DEtS species. Indeed, these molar ratios outperformed the conversion and selectivity of the 2:1 molar ratio, suggesting that the lower number of hydrogen interactions may improve SA availability for the biocatalytic reaction. Only the 1:2 TOPO:SA mixture remained heterogeneous and yielded the poorest results, 7.8 ± 2.7 DEtS yield, most likely because of the insufficient TOPO concentration. It should be noted that this yield is also underestimated, as not all the added SA is completely solubilized and cannot be accounted for in the yield calculations. Therefore, the hydrogen interactions between TOPO and SA must be finely adjusted to facilitate SA solubilization, while enabling SA availability for the biocatalytic esterification.

All reaction systems (except for 1:2 TOPO:SA) remained as homogeneous media after the biocatalytic reaction. Despite the high level of SA conversion (up to 86%), TOPO did not precipitate after cooling to room temperature, which suggests that the remaining SA and accumulated MEtS could sustain hydrogen interactions with TOPO that maintain the eutectic mixture. Alternatively, the hydrophobic behavior of ester products could also be solubilized in TOPO, consequently decreasing the melting temperature of the overall reaction system.

Since this is the first time that TOPO has been used as a reaction medium for biocatalytic reactions, enabling high dicarboxylic acid concentration, it was mandatory to demonstrate enzyme stability within this environment. To address this, the 2:1 TOPO:SA molar ratio was selected as the model reaction, with the enzyme incubated in the eutectic mixture for three consecutive days at room temperature and without stirring. Periodically, the remaining enzyme activity was tested after the addition of EtOH, revealing 100% residual activity and selectivity with respect to the original one before incubation, therefore demonstrating the excellent enzyme stability in the presence of TOPO (see Table S1).

Referring to Table 1, in neither molar ratio is the limit of 50% selectivity towards DEtS synthesis surpassed. It should be noted that biocatalytic DEtS synthesis by direct esterification of SA occurs through two sequential steps with an intermediate formation of MEtS (see Figure 1). The time-course profiles of the reactions at each TOPO:SA molar ratio revealed that this 50% selectivity is maintained throughout the incubation time (except for the 2:1 molar ratio, Figure 3), revealing the equilibrium between the two esterification reactions in all systems. Considering these profiles, both the best selectivity and the faster kinetics correspond to 1.5:1 and 1:1 molar ratios, reaching 50% conversion in 7.5 h, while for 2:1 and 1:2 TOPO:SA (mol/mol) mixtures, this parameter barely reaches 33% and 20%. These data also support that the reduced number of hydrogen-bonding interactions favors the availability of SA and MEtS for biocatalytic esterification, ultimately leading to the formation of DEtS.

### 2.2. Influence of Water Addition as Cosolvent

To improve the DEtS yield, two critical parameters were examined: the mixture viscosity and the strong hydrogen bond interactions between TOPO and SA. The systems 2:1 and 1:1 TOPO:SA molar ratios were selected as representative models for the full and partial eutectic mixtures. The viscosity measurements revealed Newtonian behavior, with the 1:1 TOPO:SA mixture exhibiting a slightly higher viscosity than the 2:1 mixture. (61.7 vs. 52.3 cP, see Figure S4). This agrees with previous reports on TOPO-based hydrophobic DESs, in which increasing the HBD content resulted in higher viscosity [25]. Thus, the superior catalytic performance observed with the equimolar mixture cannot be attributed to improved mass transfer resulting from lower viscosity and points to the number of hydrogen-bond interactions as the critical parameter governing the reaction. In this context, the addition of a small amount of water to the eutectic mixture to disrupt the hydrogen-bonding interactions between the HBD and HBA (which is a common strategy to reduce the overall viscosity) [34] may increase the availability of solubilized SA for biocatalytic esterification and enhance the overall performance. Therefore, two series of 2:1 and 1:1 TOPO:SA molar ratios (as representatives of full and partial eutectic mixtures) were prepared, adding different concentrations of water (0–20% *w*/*w*) with respect to the overall mass (accounting for EtOH mass). Water activity was determined for each system before and after the biocatalytic reaction as well as for TOPO and SA alone. TOPO and SA share a similar water activity (a_w_ = 0.36), thus obtaining the same value for both eutectic mixtures in the absence of added water (Table 2). Under these conditions, up to 44.2 or 32.4% yield is obtained with 1:1 and 2:1 TOPO:SA molar ratios, respectively. The reaction carried out with a dehydrated 1:1 TOPO:SA mixture (pre-incubated overnight with molecular sieves 13X, a_w_ = 0.15), or the addition of MS 13X to the reaction, afforded a similar yield, indicating that the eutectic mixture water content is not a limiting factor and that the use of MS 13X is therefore unnecessary to achieve efficient reaction performance. The addition of water increased water activity and resulted in heterogeneous systems for 1:1 TOPO:SA molar ratio, and biphasic systems for 2:1 TOPO:SA molar ratios at water concentrations above 5% (*w*/*w*), with a detrimental effect on both the SA conversion and DEtS selectivity (Figure S4), likely due to water-induced ester hydrolysis and the partitioning of EtOH into the aqueous phase. These results reveal the incapacity of water to disrupt the strong interactions between TOPO and SA, which is consistent with previous reports on the use of TOPO for the extraction of dicarboxylic acids from aqueous media [25,26].

### 2.3. Optimization of Reaction Conditions

To surpass the limit of 50% selectivity and improve the yield of DEtS synthesis, both the reaction temperature and EtOH concentration were optimized. Both parameters are critical, as high temperatures may provoke EtOH evaporation or dehydration to form diethyl ether [10]; meanwhile, high EtOH concentrations may lead to the biocatalyst’s deactivation by dehydration.

The biocatalytic synthesis of DEtS from the TOPO:SA 1:1 (mol/mol) mixture was evaluated at 50, 60, and 70 °C (Figure 4). No significant differences in the overall performance were observed among the temperatures tested. Therefore, 60 °C was selected as the optimal reaction temperature, as it provided the highest, albeit only slightly improved, parameters.

To further improve the DEtS yield, the impact of water removal during the reaction was investigated. However, comparable results were obtained regardless of the use of MS 13X. Neither pre-drying the eutectic mixture overnight with the desiccant, increasing the amount of MS 13X tenfold, nor adding it during the reaction resulted in any improvement in the final DEtS yield. These results suggest that the hydrophobicity and hygroscopicity of TOPO are key to controlling hydrolytic side reactions of water produced along the reaction, either by expelling water from the reaction system or by incorporating water into the hydrogen network. This eliminates the need for dehydrating agents or other costly strategies like pervaporation, vapor permeation, or reactive distillation for continuous water removal [8,35].

Subsequently, the optimal EtOH concentration required to shift the equilibrium toward DEtS formation was evaluated. Figure 5 shows the reaction parameters (conversion, selectivity, and yield) at different molar ratios of EtOH:SA. The lowest molar ratio, 2:1 EtOH:SA (corresponding to the reaction stoichiometry to synthesize DEtS), provides optimal conversion and selectivity, both increasing with EtOH concentration up to 4:1 EtOH:SA (mol/mol). Beyond this molar ratio, the high EtOH concentration has detrimental effects on enzyme stability, reducing SA conversion, although the higher availability improves the synthesis of DEtS, obtaining the highest selectivity (58%). Then, a stepwise addition was implemented to provide sufficient EtOH without the deleterious effect of high concentrations. Starting from 2:1 EtOH:SA (mol/mol), two sequential additions of EtOH were carried out at 5 and 7.5 h to finally reach a final concentration of 6:1 EtOH:SA (mol/mol). As a result, 92.0 ± 2.3% conversion and 60.0 ± 4.4% selectivity were achieved, thereby increasing the DEtS yield by up to 108% and 48% compared with the DEtS yield obtained with 2:1 and 6:1 EtOH:SA molar ratios, respectively.

### 2.4. Product Extraction

As previously described, the homogeneous media obtained at the end of the reaction indicates the stabilization of the eutectic mixture despite the high SA conversion into the products. This indicates that TOPO maintains hydrogen interactions with unreacted SA and the free carboxylic acid in the MEtS product.

Attempts to disrupt the hydrogen bonds by adding water, NaCl-saturated water, or increasing the pH to deprotonate SA and MEtS were unsuccessful. The similar miscibility of TOPO and DEtS in the solvents evaluated also hindered the development of a liquid-liquid extraction strategy for product recovery. Only DMSO was identified as a suitable extractant, acting as an antisolvent inducing TOPO crystallization. Accordingly, two volumes of DMSO were added to the reaction mixture after removal of the biocatalyst. After a short incubation without agitation, the crystallization of TOPO became apparent, facilitating its removal by centrifugation at 3000 rpm for 10 min through a 3000 MWCO filter (Figure 6), further improved by a second filtration through a 0.2 µm pore size. Thus, ^31^P-NMR analyses revealed that after the first centrifugation step, 96% of the TOPO content was removed from the reaction system, and it was completely removed after the second centrifugation step. This permitted recovery of crystalline TOPO retained in the centrifugal filter for reuse, and all the reaction components dissolved in the DMSO phase were completely free of TOPO.

### 2.5. Sustainable Assessment of SA Esterification

The current framework of sustainable chemistry calls for the development of efficient, safe, and waste-minimizing strategies. Although rDES-based technologies have been shown to enhance process sustainability by reducing the need for large volumes of additional solvents, which are often a major source of waste [36], this improvement must be substantiated through quantitative data obtained from green metrics analyses.

Furthermore, to highlight the innovation and advantages of the proposed TOPO-based rDES, two strategies of biocatalytic DEtS synthesis have been selected from the literature for comparison (see Table 3).

Biocatalytic esterification of SA to yield DEtS has been scarcely reported, mainly due to the low solubility of SA in hydrophobic media, which has limited the identification of suitable strategies for comparative analysis. The two strategies selected for comparison rely on the use of either a hydrophobic organic solvent or a biphasic aqueous/alcohol system to enable the solubilization and esterification of SA. Notably, the concentration of solubilized SA in these approaches is 1 to 2 orders of magnitude lower than the reported strategy based on rDES with TOPO. It should also be noted that both strategies evaluate reaction performance in terms of SA conversion. In the study by Abdul Rahman et al. [37], conversion was monitored by FTIR and TLC analyses, while the diester product was barely detected by GC-MS spectrometry. Delhomme et al. [31] compared the chemical and biocatalytic synthesis of DEtS, with the diester product being detected only when chemical catalysis was employed. Therefore, the assumption of 100% selectivity in these approaches should be regarded with caution.

According to the results, the biocatalytic reaction performs better in hydrophobic media as expected, enabling the best conversions. However, this comparative analysis clearly illustrates that higher productivity does not necessarily translate into greater sustainability. Thus, although the best productivity is achieved in hexane, this strategy also generates a large amount of waste (9.5 kg/kg product). Moreover, hexane accounts for 61% of the waste and is a flammable, toxic, corrosive, and hazardous solvent classified as undesirable by most solvent selection guides [38]. In contrast, the use of rDES technology enables optimal conversion and selectivity with the lowest waste (1.8 kg waste/kg product). This can be attributed to the absence of a conventional solvent, as only a small amount of TOPO is required to solubilize high concentrations of SA, being subsequently recovered for reuse and therefore not contributing to waste. By contrast, the strategy reported by Delhomme et al. [31] generated the largest amount of waste (73.8 kg/kg product), mainly originating from the aqueous phase and excess alcohol (see mass-balance of reactions in Table S3).

The representation of Andraos pentagon [39] using the most representative mass-based metrics further demonstrates the superior performance of the rDES strategy (Figure 7). Indeed, this approach yields the polygon closest to the ideal values highlighted in green. It should be noted that this analysis is restricted to the upstream stage, as the other strategies do not report downstream processing or lack sufficient quantitative information regarding the volume of solvents used, thereby hampering the analysis.

Regardless of the nature of generated waste, the ultimate fate is always the complete oxidation to CO_2_, which can be calculated with the Total Carbon Release (TCR) parameter [40]. TCR is defined as the mass of carbon dioxide produced per kilogram of product. TCR is calculated from the Process Mass Intensity (PMI), a metric closely related to the E-factor through the relationship PMI = E-factor + 1, since PMI includes the mass of products in the calculations [41]. Therefore, the lowest achievable value of PMI is 1 (meaning that all reagents are incorporated in the products) and is more suitable than the E-factor for estimating TCR, as it also accounts for the contribution of the products to CO_2_ release. This metric can be easily determined using the ACS PMI calculator worksheet [42], which discriminates between organic and aqueous sources of waste. This distinction is fundamental for the accurate determination of TCR, since the energy requirements associated with their incineration, assuming the worst-case scenario of waste treatment, differ substantially.

In this regard, the PMI determined using the ACS calculator agrees with the calculated E-factor, and the resulting TCR values further support the lower CO_2_ emissions enabled by the rDES technology developed in this work. These findings are particularly relevant in the context of the urgent need to reduce greenhouse gas emissions and limit the accumulation of CO_2_ in the atmosphere.

## 3. Materials and Methods

### 3.1. Materials

Commercial immobilized *Candida antarctica* lipase B (Novozym^®^ 435, EC 3.1.1.3) was provided by Novozymes S.A. (Madrid, Spain). Sigma (Madrid, Spain) provided the following reagents: succinic acid (≥99.5%), a standard of DEtS (99%), trioctylphosphine oxide (TOPO, 99%), the desiccant agent MS 13X (270 mg H_2_O/g adsorption capacity), and dimethyl sulfoxide (DMSO-*d*_6_). EtOH of HPLC grade (99.9% purity) was provided by PanReac AppliChem (Barcelona, Spain).

### 3.2. Biocatalytic Synthesis of DEtS in Eutectic Mixtures

To obtain the eutectic mixture, different molar ratios of TOPO and SA (2:1; 1.5:1, 1:1, and 1:2 mol/mol) were magnetically stirred at 340 rpm and heated at 70 °C for 1 h, in 4–10 mL glass vials.

The esterification reaction proceeded after the addition of EtOH at different molar ratios (6:1, 4:1, 2:1 EtOH:SA mol/mol) and the biocatalyst N435 (33 mg/mmol SA). Alternatively, the effect of adding the desiccant agent MS13 X (33 mg/mmol SA) was also evaluated. Incubation was conducted in an orbital shaker at 60 °C, 250 rpm, for 20–24 h.

The synthesis of DEtS follows two sequential steps of esterification with the intermediate formation of MEtS according to Figure 1. To analyze the biocatalytic performance of both products, we obtained time–course profiles by withdrawing reaction aliquots at regular intervals and analyzing them by ^1^H-NMR.

To test N435 stability in TOPO-based reaction mixtures, different vials were prepared containing a mixture of 2:1 (mol/mol) TOPO:SA. The biocatalyst was added to all vials simultaneously, which were subsequently incubated at room temperature without stirring. On each sampling day, the residual enzyme activity was measured in a separate vial following the addition of EtOH and an 8-h incubation period at 60 °C.

### 3.3. FTIR Analysis

An ATR-FTIR-4700 spectrometer (JASCO Analytical Instruments, Easton, PA, USA) with 0.4 cm^−1^ resolution and a measurement range of 4000–400 cm^−1^ was used to determine the bands corresponding to the functional groups of substrates. The eutectic mixture was confirmed by the shift of the vibrating band of TOPO P=O stretching from 1144.55 cm^−1^ to 1125.26 cm^−1^. This indicated a weakness in the P=O interaction as a result of proton acceptance. Equally, C=O stretching in SA provoked the shift of the vibrating band from 1681.62 cm^−1^ to 1711.51 cm^−1^.

### 3.4. Differential Scanning Calorimetry (DSC)

Samples (1:1 and 2:1 TOPO:SA molar ratios) were analyzed using a TA Instruments DSC 2920 differential scanning calorimeter (New Castle, DE, USA). 6 mg of the sample was weighed, encapsulated in an aluminum pan, and hermetically sealed. The temperature program consisted of a heating ramp from room temperature to approximately 250 °C at a constant heating rate of 10 °C/min under a nitrogen atmosphere.

### 3.5. Viscosity Determinations

TOPO, 1:1 and 2:1 TOPO:SA (mol:mol) mixtures were selected to determine their viscosity using a Modular Compact Rheometer (MCR 102, Anton Paar, Madrid, Spain) with a cone and plate geometry. A shear rate from 1 to 100 s^−1^ was used, collecting a total of 100 measurements for each sample within a range of 200 s at 60 °C (the reaction temperature).

### 3.6. ^1^H-NMR and ^31^P-NMR Analysis

Substrate and product characterization was carried out by ^1^H-NMR analyses conducted on a Bruker Avance 400 MHz spectrometer (Bruker BioSpin AG, Fällanden, Switzerland).

Aliquots of 20 µL from the liquid phase of the reaction mixtures were mixed with DMSO-*d*_6_ containing *tert*-butanol (*t*-BuOH) as an internal standard (100 mM final concentration). After thorough mixing, the mixture was centrifuged at 15,000 rpm at 5 °C for 5 min to separate the solid TOPO. The assignment of protons follows the designation in Figure S3.

^1^H-NMR δ (ppm): EtOH: H_A_ 1.05 (t, 3H), H_B_ 3.44 (dq, 2H), Hc 4.36 (s, 1H). SA: H_A_ 2.39 (s, 4H). *tert*-butanol: H_A_ 1.10 (s, 9H), H_B_ 4.19 (s, 1H). Monoethyl succinate (MEtS): H_A_ 1.17 (t, 3H), H_B_ 4.05 (q, 2H), H_C_ and H_D_ 2.46 (m, 4H, indistinguishable). Diethyl succinate (DEtS): H_A_ 1.17 (t, 6H), H_B_ 4.05 (q, 4H), H_C_ 2.53 (s, 4H).

Conversion of SA into the ester products and selectivity towards DEtS were calculated from the ^1^H-NMR spectra, using the areas of protons that specifically identify each compound, according to the following equations:

(1) $$\mathrm{Conversion}\;\left(\%\right)=\;\frac{H_{C}\;\mathrm{DEtS}+(H_{C}\;\mathrm{MEtS}+\;H_{D\;}\mathrm{MEtS})\;}{H_{B}\;\mathrm{SA}+\;H_{C}\;\mathrm{DEtS}+(H_{C}\;\mathrm{MEtS}+\;H_{D}\;\mathrm{MEtS})}$$

(2) $$\mathrm{Selectivity}\;\left(\%\right)=\frac{H_{C}\;\mathrm{DEtS}}{H_{C}\;\mathrm{DEtS}+(H_{C}\;\mathrm{MEtS}+H_{D}\;\mathrm{MEtS})}$$

After the biocatalytic reaction, the reaction mixture was diluted with two volumes of DMSO. This antisolvent-induced TOPO crystallization was further separated by two sequential centrifugation steps using 3000 MWCO and 0.2 µm pore-size filters. ^31^P-NMR analyses were carried out to determine residual TOPO in the extracted products. 45 µL of the reaction mixture or the extracted products in the DMSO phase were mixed with 460 µL acetone-*d*_6_ containing 20 µL LI [P_6,6,6,14_][PHOS] as an internal standard. The residual TOPO content was determined relative to a sample of the reaction mixture collected before DMSO addition. The measured values were corrected for the dilution resulting from DMSO addition.

### 3.7. Green Metrics Analysis

To determine the mass efficiency of the proposed strategy for SA esterification, the following green metrics were selected: Conversion (Conv), Atom Economy (AE), inverse of Stoichiometric Factor (1/SF), Mass Recovery Parameter (MRP), Reaction Mass Efficiency (RME), and E-factor (E-f). The equations used are summarized in Table S2 [22].

The ACS PMI calculator [42] was used to determine the Process Mass Intensity (PMI). Total Carbon Release (TCR) [40] was calculated from the PMI values for organic or aqueous waste according to Equation (S8) in Table S2.

## 4. Conclusions

Biocatalytic synthesis of DEtS by direct esterification of SA is hampered by low SA solubility in hydrophobic media, enzyme inactivation at high EtOH concentrations, and thermodynamic equilibrium. Consequently, conventional processes generally rely on using succinic anhydride or metal- or ion-exchange resin-based catalysts in aqueous or organic media.

The incorporation of SA as a counterpart of a rDES with TOPO rules out the need for additional solvents, providing optimal conditions (pH, hydrophobic environment, high substrate concentration, water activity control) for the biocatalytic esterification with EtOH. To the best of our knowledge, this is the first report demonstrating the suitability of TOPO as a reaction medium for biocatalytic transformations.

Although the reaction thermodynamic equilibrium and the strong interactions between TOPO and SA or MEtS remain significant limitations to achieving the highest yield, the limit of 50% selectivity could be overcome through the sequential addition of EtOH, finally obtaining 56% yield in just 20 h at 60 °C and with only a slight excess of EtOH with respect to SA. Furthermore, an efficient and simple downstream strategy was designed based on the addition of DMSO as an antisolvent, inducing TOPO crystallization and enabling the recovery of the ester products. In this context, the TOPO-based rDES technology provides an optimal balance between yield and process sustainability, avoiding the use of non-recommended solvents and reducing waste.

Although further work is still needed to improve the proposed system, particularly enhancing DEtS synthesis and demonstrating the reusability of TOPO and the biocatalyst, the strategy reported herein represents a promising platform for the sustainable biocatalytic synthesis of DEtS.

## Figures and Tables

**Scheme 1:**
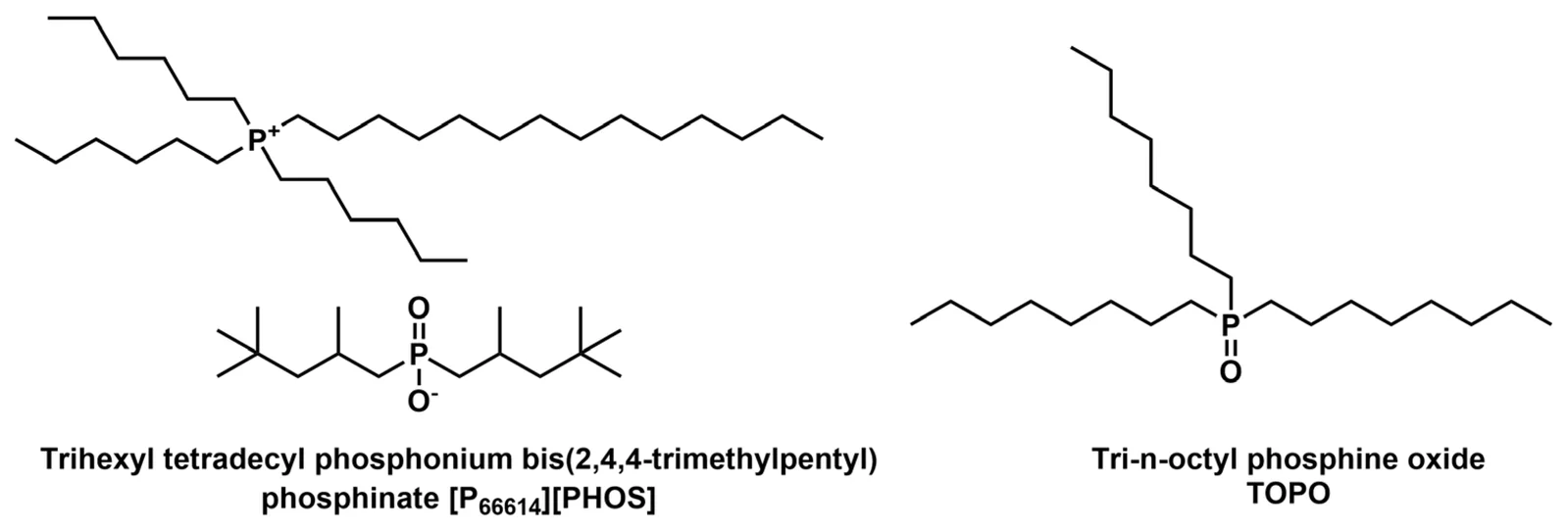
Structures of TOPO and the IL [P_6,6,6,14_][PHOS].

**Figure 1:**
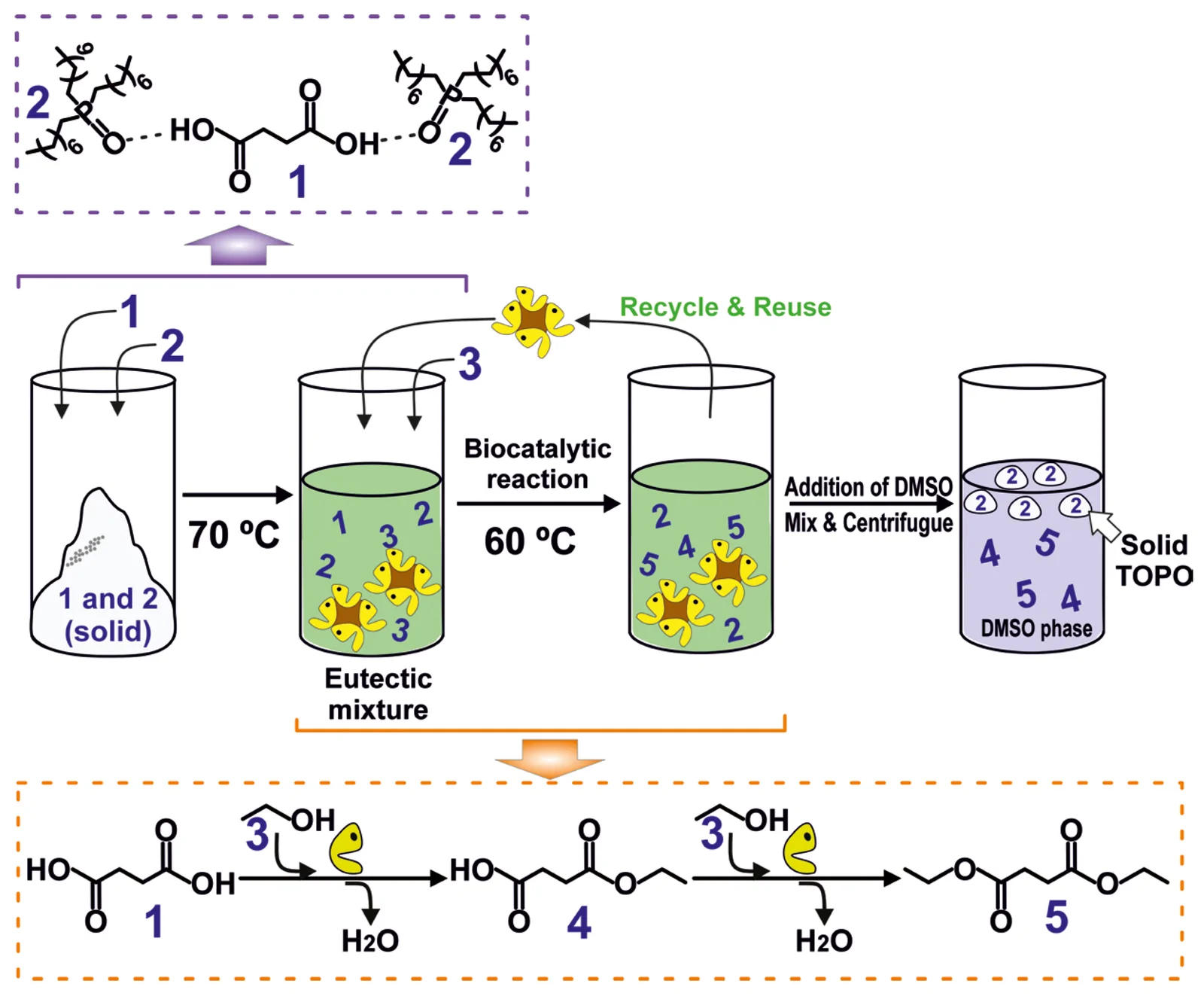
Representative scheme for diethyl succinate synthesis in hydrophobic trioctylphosphine oxide (TOPO)-based rDES. After heating SA (**1**) and TOPO (**2**) at 70 °C, hydrogen bond interactions are established, leading to a monophasic liquid system. Thus, the addition of the commercial immobilized biocatalyst N435 and EtOH (**3**) promotes SA esterification into mono- and di-ethyl succinate species (**4** and **5**, respectively). Finally, after removing the enzyme by simple filtration and DMSO addition, the ester products are solubilized in the DMSO phase, while crystallized TOPO can be easily removed.

**Figure 2:**
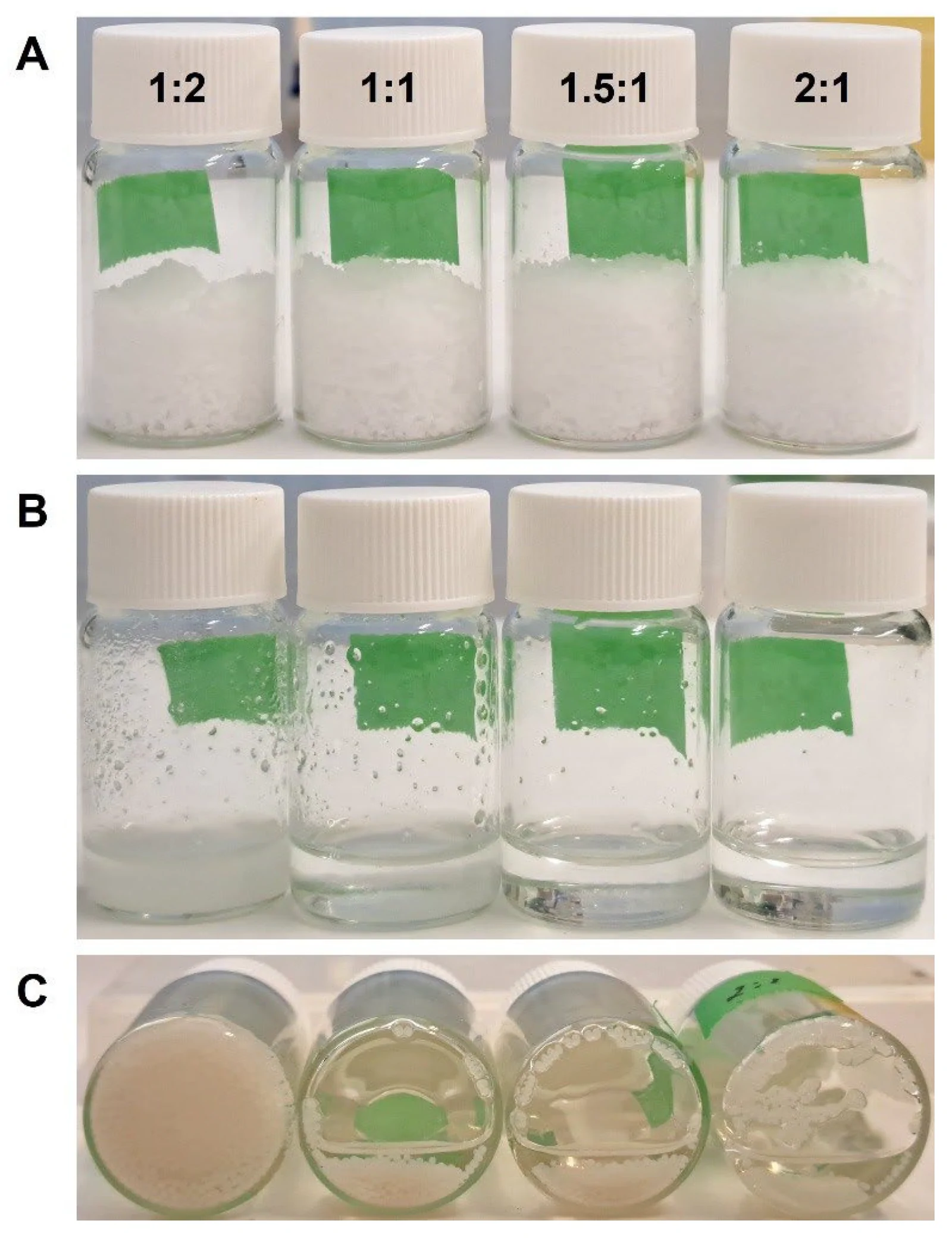
Identification of TOPO:SA eutectic mixture. (**A**) Mixtures of different molar ratios of TOPO (m.p. 56.3 °C) and SA (m.p. 192.3 °C) at room temperature (R.T.). (**B**) Verification of the eutectic mixture after heating at 70 °C for 1 h and cooling to R.T. Only the 2:1 TOPO:SA molar ratio completely solubilizes SA. (**C**) Reaction mixtures containing the immobilized enzyme after the esterification reaction at 60 °C for 20 h, 4:1 EtOH:SA molar ratio, 33 mg N435/mmol SA. All mixtures evolved into homogeneous systems, except for the 1:2 TOPO:SA mixture, in which SA formed a solid layer at the bottom of the vial, entrapping the immobilized biocatalyst N435. For these pictures, the amounts of TOPO and SA were adjusted to obtain similar volumes while maintaining the desired ratios to facilitate visual comparison.

**Figure 3:**
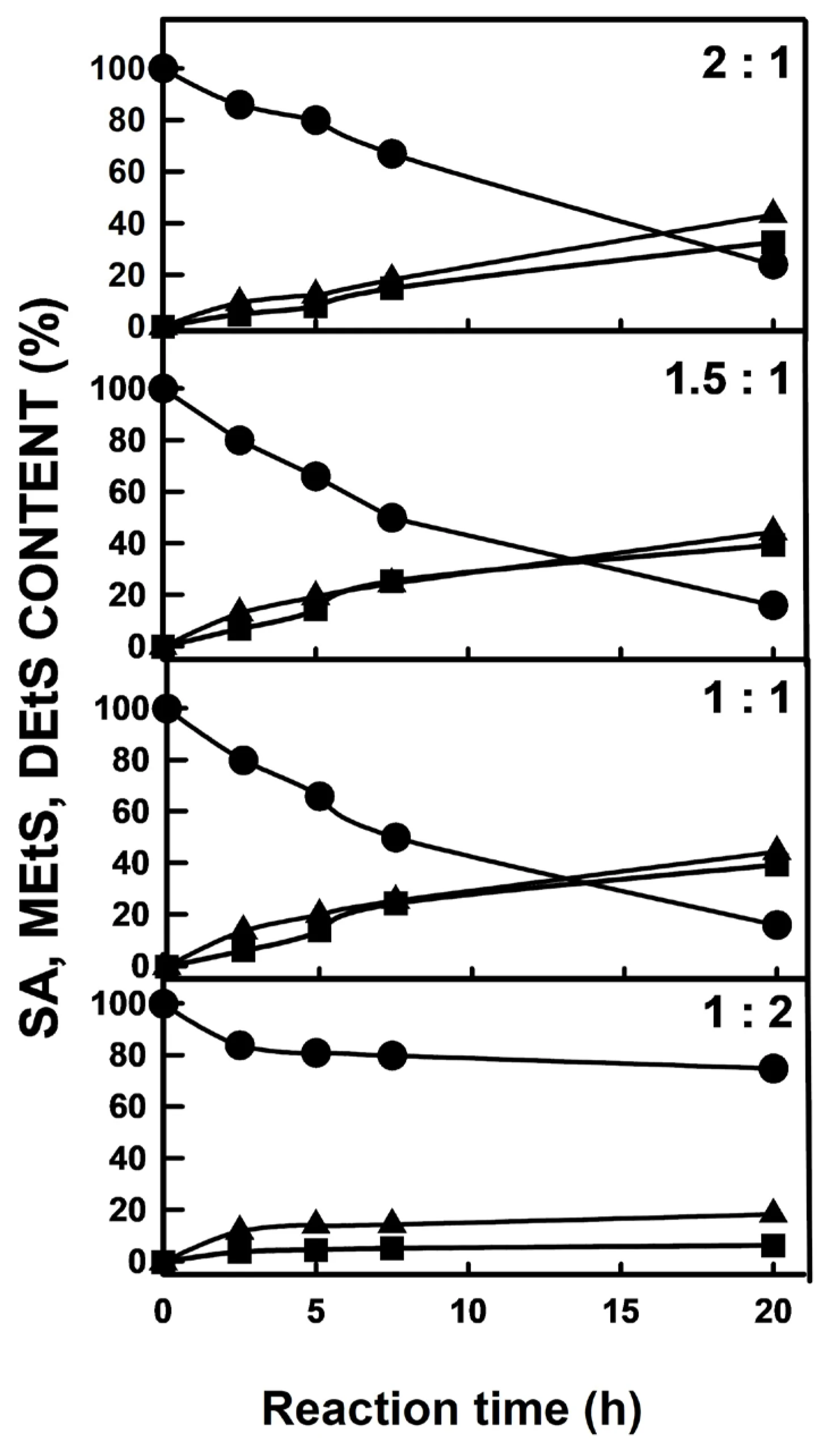
Time-course profiles of diethyl succinate synthesis in rDES with different TOPO:SA molar ratios. Legend: (●) SA; (▲) MEtS; (■) DEtS. Conditions: 5 mmol SA, 20 mmol EtOH, 33 mg N435/mmol SA, 60 °C, 250 rpm, 20 h.

**Figure 4:**
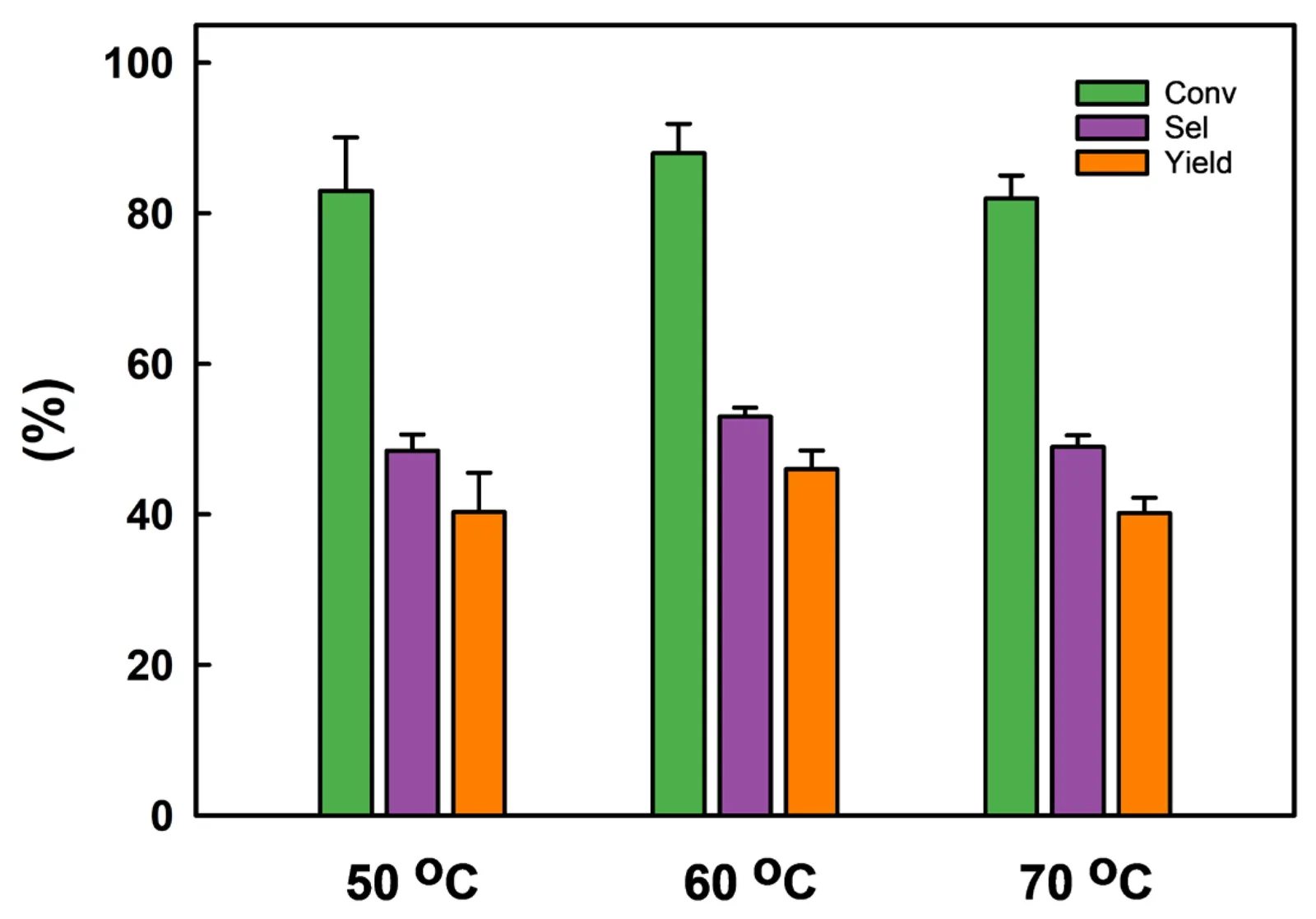
Influence of reaction temperature in the biocatalytic synthesis of DEtS. Reaction mixtures 1:1 TOPO:SA were prepared using 5 mmol each. The addition of 20 mmol EtOH and 3 mg_N435_/mmol SA started the reaction, which was incubated at the selected temperature for 20 h at 250 rpm. Conv: Conversion (green bar), Sel: Selectivity (purple bar), diethyl succinate Yield (orange bar). Data obtained from three replicates.

**Figure 5:**
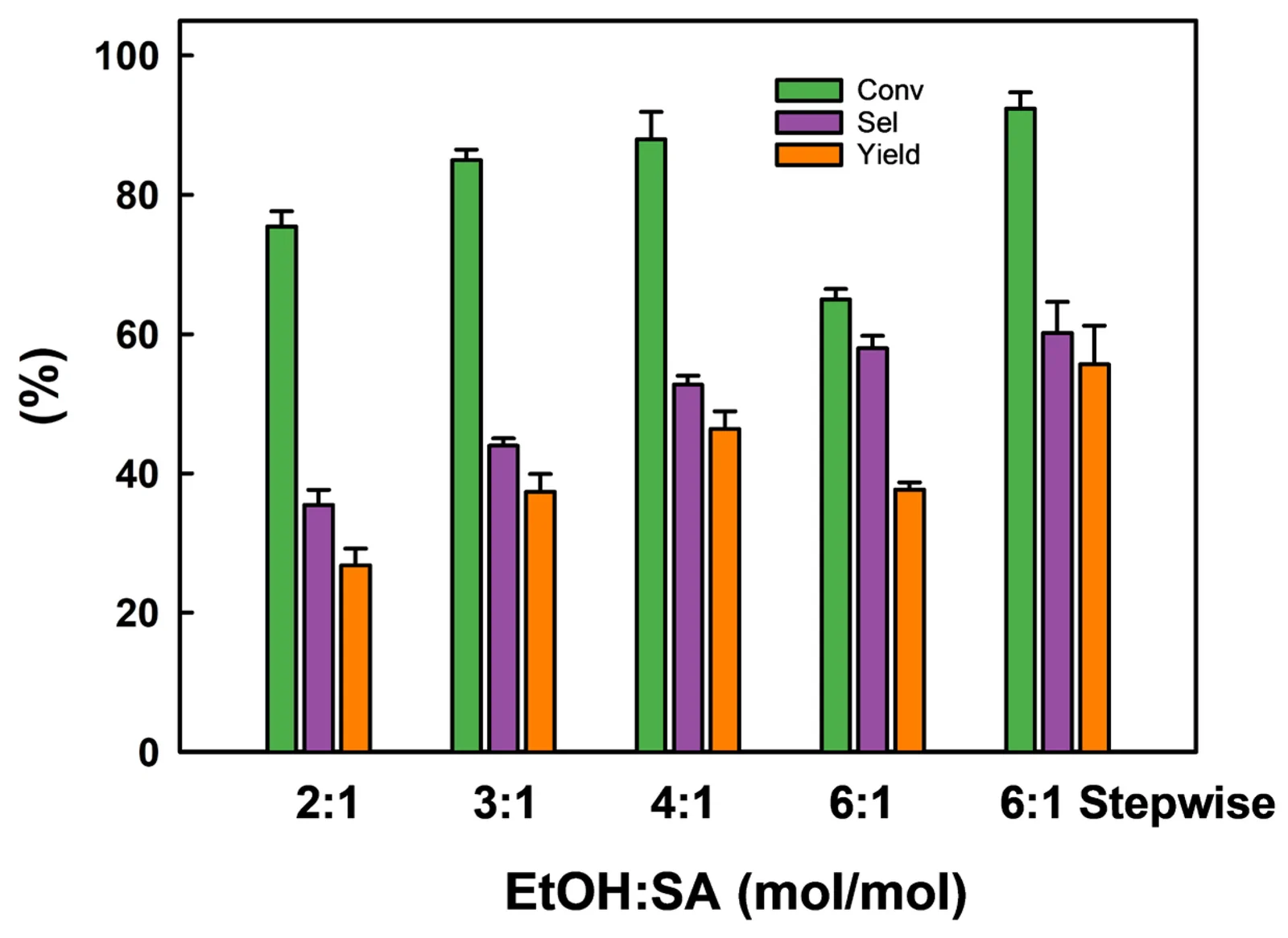
Impact of EtOH concentration on diethyl succinate synthesis. Reaction mixtures were prepared using 3 mmol SA. The addition of 33 mg_N435_/mmol SA started the reaction, which was incubated at 60 °C for 20 h at 250 rpm. Conv: Conversion (green bar), Sel: Selectivity (purple bar), DEtS Yield (orange bar). Data obtained from three replicates.

**Figure 6:**
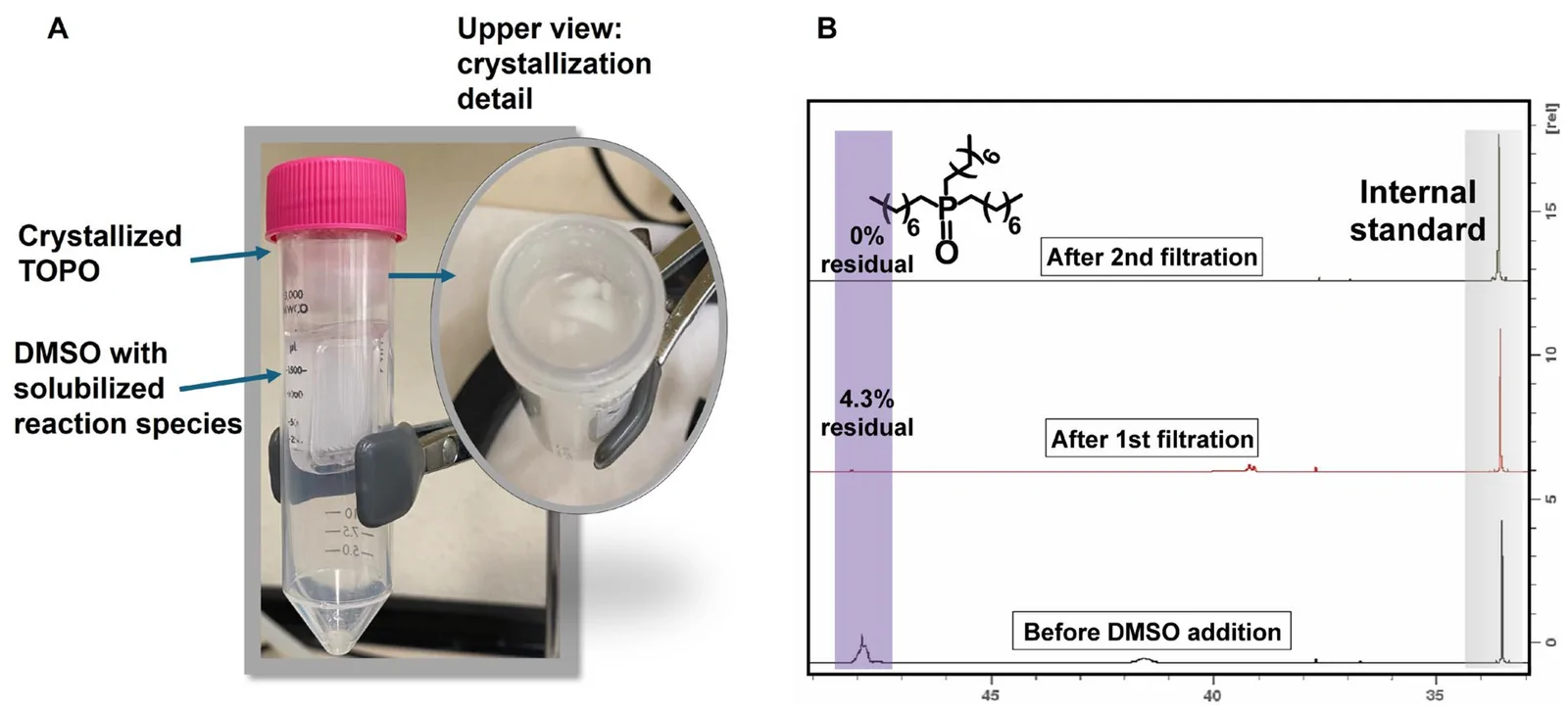
Product extraction and TOPO recovery. (**A**) Filtration of crystallized TOPO in the presence of DMSO. (**B**) ^31^P-NMR of the reaction mixture and the two filtered supernatants. 20 µL of the IL [P_6,6,6,14_][PHOS] were used as internal standard.

**Figure 7:**
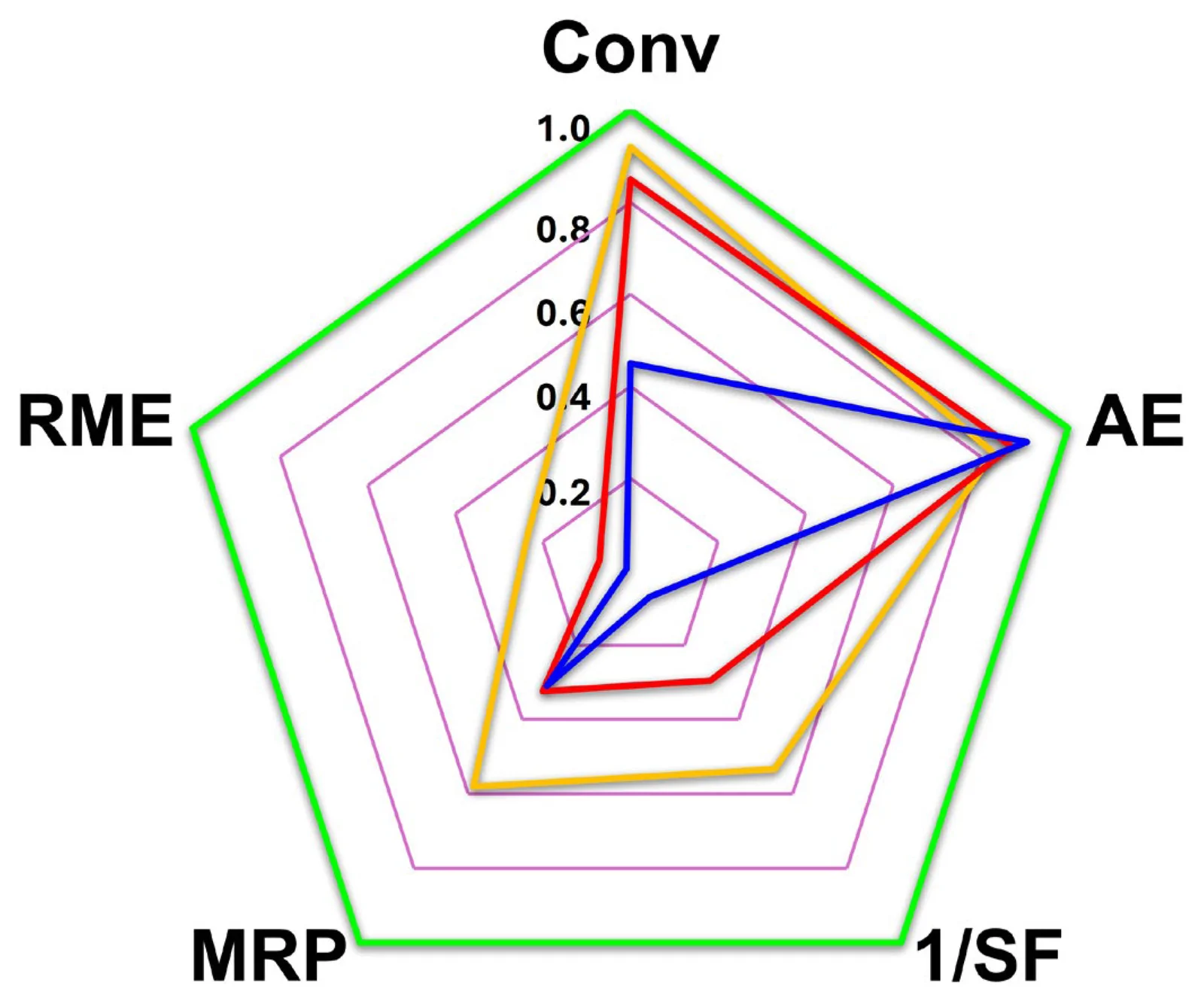
Andraos representation of calculated mass-based metrics. This work (orange polygon); Abdul Rahman et al. [37] (red polygon); Delhomme et al. [31] (blue polygon). The green frame indicates the highest sustainability.

**Table 1:** Influence of the TOPO:SA molar ratio on the biocatalytic conversion and selectivity in DEtS synthesis. Conditions: 5 mmol SA, 20 mmol EtOH, 33 mg N435/mmol SA, 60 °C, 250 rpm, 20 h. The pH of the mixture after the biocatalytic reaction is also shown.

Entry	TOPO:SA (mol/mol)	Conversion (%)	Selectivity (%)	Yield (%)	pH
1	2:1	79.7 ± 4.0	43.0 ± 1.0	34.2 ± 1.4	3.9
2	1.5:1	80.5 ± 3.3	47.0 ± 1.4	37.8 ± 1.7	3.7
3	1:1	85.8 ± 3.4	51.6 ± 3.5	44.2 ± 3.4	3.7
4	1:2	27.9 ± 3.0	27.7 ± 7.0	7.8 ± 2.7	3.1
5	0:1	2.8 ± 0.5	n.d.	n.d.	n.d.

n.d.: not detected.

**Table 2:** Influence of water added as a cosolvent on the biocatalytic yield. Two series of DESs with TOPO:SA molar ratios of 1:1 and 2:1 were prepared using 1 mmol of SA. Water was added to each mixture to obtain final concentrations ranging from 0 to 20% (*w*/*w*) relative to the total reaction mass, including EtOH. The reactions were initiated by adding EtOH (EtOH:SA molar ratio of 4:1) and N435 (33 mg/mmol SA), followed by incubation at 60 °C for 20 h.

H_2_O (%)	1:1 TOPO:SA mol/mol			2:1 TOPO:SA mol/mol		
	a_w_ t_0_	a_w_ t_24 h_	Yield (%)	a_w_ t_0_	a_w_ t_24 h_	Yield (%)
0	0.38	0.53	44.2	0.36	0.69	32.4
2	0.50	0.72	39.0	0.76	0.95	21.3
5	0.78	0.88	3.4	0.89	0.99	3.1
10	0.95	0.93	2.0	0.89	0.99	1.6
20	0.97	0.99	1.9	0.97	0.99	2.4

**Table 3:** Reaction conditions and green metrics of selected strategies.

	This Work	Abdul Rahman et al. [37]	Delhomme et al. [31]
**Reaction medium (mL)**	rDES (1.56) TOPO:SA 1:1 mol/mol	Hexane (5)	Water (5)
**Alcohol (mmol)**	Ethanol (18)	Oleoyl alcohol (7.8)	1-Octanol (30)
**SA:Alcohol (mol/mol)**	1:6	1:7.8	1:40
SA (M) *	1.15	0.13	0.08
**Catalyst (mg)**	N435 (99)	N435 (20)	N435 (40)
**Temperature (°C)**	60	41.1	70
**Time (h)**	20	4.5	24
**Conversion (%)**	92	85	47
Selectivity (%) **	60	100	100*
**Productivity (µmol/mg_N435_)**	27.9 ± 0.5	42.5	8.8
**E-factor**	1.8	9.5	73.8
**PMI_org_**	2.7	10.5	33.4
**PMI_aq_**	0.0	0.0	41.3
**TCR (kg CO_2_/kg prod)**	6.2	24.2	102.8

* SA concentration in the overall reaction mixture, including alcohol. ** 100% selectivity is assumed, although it is not explicitly stated in the manuscript.

## Data Availability

The original contributions presented in this study are included in the article and Supplementary Materials. Further inquiries can be directed to the corresponding authors.

## Supplementary Materials

The following supporting information can be downloaded at: https://www.mdpi.com/article/10.3390/molecules31193402/s1, Figure S1: FTIR analyses of TOPO:SA eutectic mixtures. (A). FTIR spectra of TOPO, SA and the mixture 2:1 TOPO:SA. Hydrogen bond interactions are confirmed by the shift of vibrating bands corresponding to the functional groups involved. (B). FTIR spectra of the liquid phases of mixtures at different molar ratios TOPO:SA; Figure S2: DSC analysis of standards and eutectic mixtures. This analysis confirms the melting temperature of TOPO (56.3 °C) and SA (192.3 °C) alone. The absence of a peak in the 2:1 TOPO:SA mixture indicates that the reaction mixture is liquid in the range between 40–220 °C, then confirming the eutectic mixture. In the 1:1 molar ratio, the broad peak may correspond to the small fraction of insolubilized SA in the eutectic mixture; Figure S3: ^1^H-NMR analysis. Representative ^1^H-NMR spectrum of the reaction medium containing SA, ester species, EtOH and *tert*-butanol as internal standard, as well as the assignation of protons of the reaction species. TOPO signals are observed due to the presence of TOPO particles in suspension in the NMR tube; Figure S4: Viscosity of TOPO and eutectic mixtures. The linear relationship between the shear rate (1/s) and viscosity (cP) reveals a Newtonian behavior of the eutectic mixtures; Figure S5: DEtS and MEtS synthesis in presence of water as cosolvent. (A). 1:1 TOPO: SA molar ratio with different amounts of added water. (B). 2:1 TOPO: SA molar ratio with different amounts of added water. The eutectic mixtures can stand up to 2% water. From 5% water content (0.8–0.9 a_w_) both conversion and selectivity are dramatically affected although a 10–20% synthesis can yet be observed; Table S1: Determination of N435 stability in TOPO:SA 2:1 mol/mol eutectic mixture; Table S2: Green metric parameters equations; Table S3: Mass-balance of strategies selected for sustainability comparison.

## Abbreviations

The following abbreviations are used in this manuscript:

AE	Atom economy
Conv	Conversion
DEtS	Diethyl succinate
DSC	Differential scanning calorimetry
E-f	Environmental factor
EtOH	Ethanol
HBA	Hydrogen bond acceptor
HBD	Hydrogen bond donor
MEtS	Monoethyl succinate
MRP	Material recovery parameter
PMI	Process mass intensity
rDES	Reactive deep eutectic solvent
RME	Reaction mass efficiency
SA	Succinic acid
Sel	Selectivity
SF	Stoichiometric factor
TCR	Total carbon release
TOPO	Trioctylphosphine oxide
