# Supplementary material for: Biocatalytic Synthesis of Diethyl Succinate in Hydrophobic Trioctylphosphine Oxide (TOPO)-Based Reactive DES
Source: Molecules. 2026 Sep 24;31(19):3402. doi: 10.3390/molecules31193402 (PMC13665810; doi:10.3390/molecules31193402)
Supplement: Supplementary file 1 [file molecules-31-03402-s001.zip › molecules-4580212-supplementary.pdf]

SUPPLEMENTARY MATERIALS

Biocatalytic Synthesis of Diethyl Succinate in Hydrophobic Trioctylphosphine Oxide (TOPO)-Based Reactive DES

Susana Nieto *, Francisco J. Ruiz, Juan Martinez-Garcia, Rocio Villa, Jairton Dupont and Pedro Lozano*

Departamento de Bioquímica y Biología Molecular "B" e Inmunología. Facultad de Química. Univer-sidad de Murcia, E-30.100, Murcia, Spain.;

* Correspondence: SN: susanani@um.es; PL: plozanor@um.es

Table of Contents

N435 stability in TOPO:SA eutectic mixtures **S2**
FTIR analysis **S2**
Differential scanning calorimetry analyses **S4**
^{1}H-NMR analysis of upstream **S5**
Viscosity determinations **S6**
Influence of water addition as cosolvent **S7**
Green Metrics analysis **S8**

N435 stability in TOPO:SA eutectic mixtures

To test N435 stability in TOPO-based reaction mixtures, different vials were prepared containing a mixture 2:1 (mol/mol) TOPO :SA. The biocatalyst was added to all vials simultaneously, which were subsequently incubated at room temperature without stirring. On each sampling day, a vial was selected to determine the residual enzyme activity after the addition of EtOH and incubation at 60 °C for 8 hours.

Table S1. Determination of N_{435} stability in TOPO:SA 2:1 mol/mol eutectic mixture.

**2:1 TOPO:SA mol/mol**					
**Entry**	**Incub. days**	**Conv (%)**	**Sel (%)**	**Residual Conv (%)**	**Residual Sel (%)**
**1**	**0**	52	48	100	100
**2**	**1**	40	41	77	85
**3**	**2**	54	47	104	98
**4**	**3**	57	48	110	100

Conditions: 5 mmol SA, 20 mmol EtOH, 33 mg N_{435} / mmol SA, 60 °C, 250 rpm, 8 h.

Incub: Incubation; Conv: Conversion; Sel: Selectivity

Note that the enzyme retains its initial performance (day 0). The lower value observed for entry 2 may be attributed to experimental variability.

FTIR analysis

An ATR-FTIR-4700 spectrometer (JASCO Analytical Instruments, Easton, PA, EE.UU.) with 0.4 cm-1 resolution and a range of measurement 4000 - 400 cm^{-1} was used to determine the vibrating bands corresponding to the functional groups of substrates. Spectra were obtained for TOPO and SA standards and mixtures obtained with different molar ratios.

Figure S1A shows the individual spectrum of TOPO, SA and the eutectic mixture achieved with 2:1 TOPO:SA molar ratio, as representative example. The eutectic mixture is confirmed by the shift of the vibrating band of TOPO P=O stretching from 1144.55 cm^{-1} to 1125.26 cm^{-1}, indicating the weakness in this interaction as results of the proton acceptance. Equally, C=O stretching in SA provoked the shift of the vibrating band from 1681.62 cm^{-1} to 1711.51 cm^{-1}.

The liquid phases of all molar ratios tested in section 2.1 were analyzed by FTIR revealing the same shifts (Figure S1B). Consequently, the eutectic mixture between TOPO and SA was achieved in all cases at least partially. This indicates that the insolubilized SA is due to a deficit of TOPO.

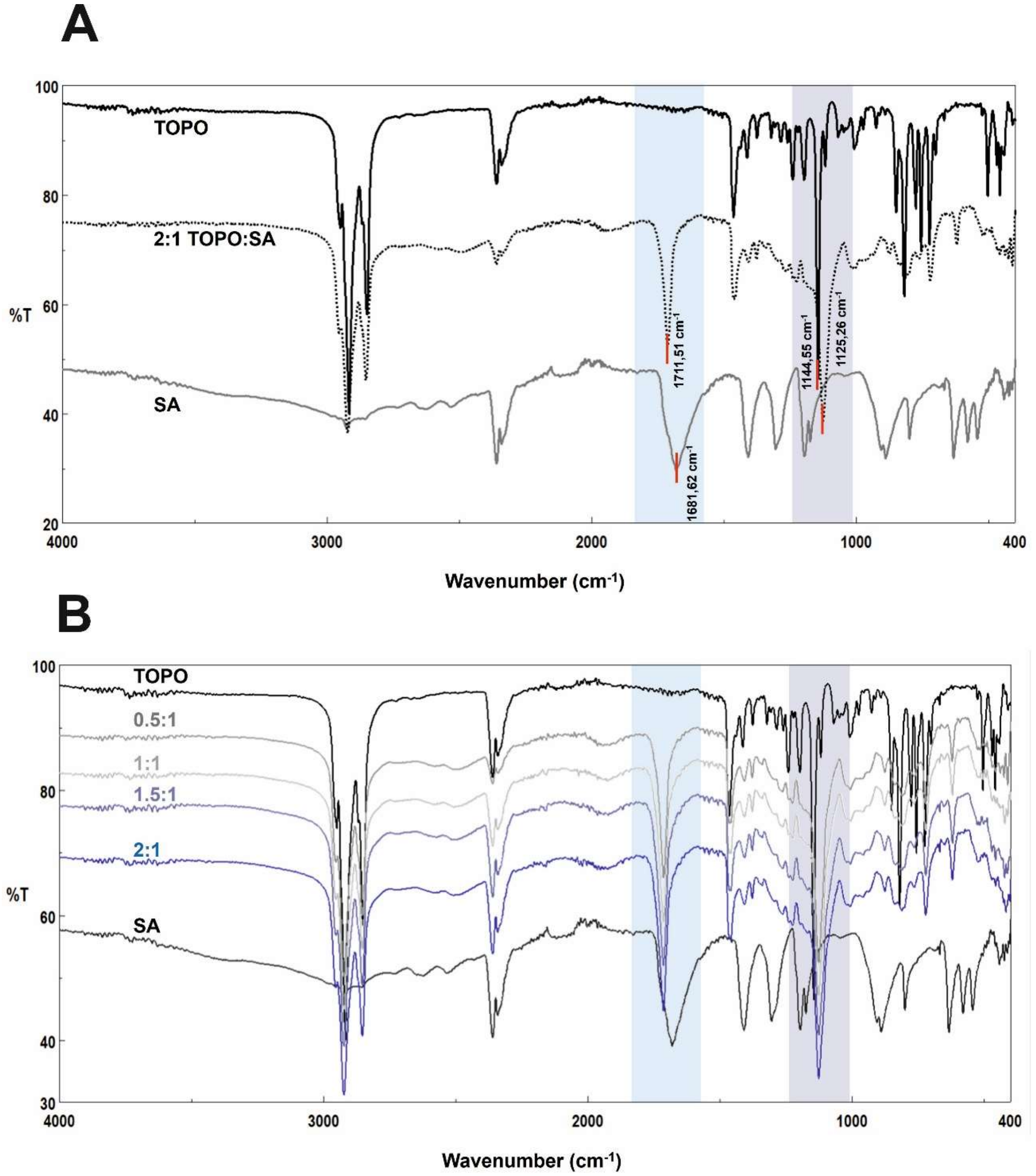

Figure S1. FTIR analyses of TOPO:SA eutectic mixtures. (**A). **FTIR spectra of TOPO, SA and the mixture 2:1 TOPO:SA. Hydrogen bond interactions are confirmed by the shift of vibrating bands corresponding to the functional groups involved. **(B).** FTIR spectra of the liquid phases of mixtures at different molar ratios TOPO:SA.

Differential scanning calorimetry analyses.

Samples (1:1 and 2:1 TOPO: SA molar ratios) were analyzed using a TA Instruments DSC 2920 differential scanning calorimeter. 6 mg of each sample were weighed, encapsulated in an aluminum pan and hermetically sealed (**Figure S2**). The temperature program consisted of a heating ramp from room temperature to approximately 250 °C at a constant heating rate of 10 °C/min under a nitrogen atmosphere.

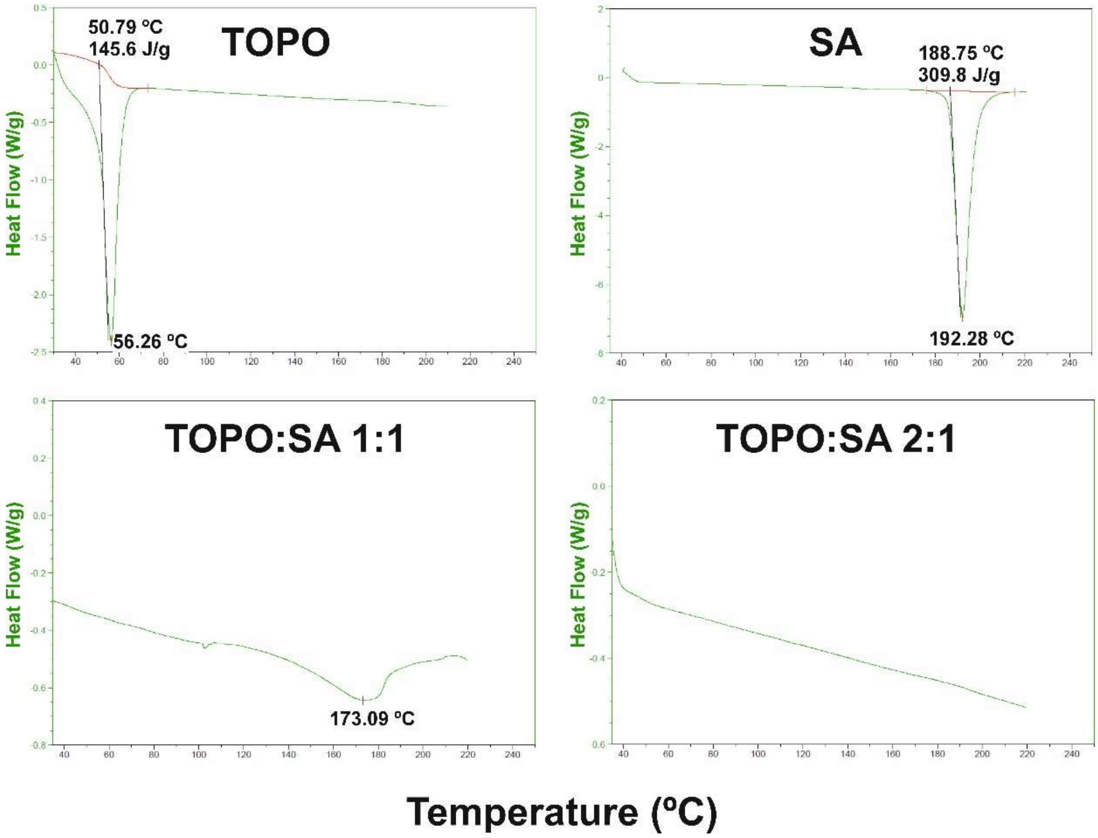

Figure S2. DSC analysis of standards and eutectic mixtures. This analysis confirms the melting temperature of TOPO (56.3 °C) and SA (192.3 °C) alone. The absence of a peak in the 2:1 TOPO:SA mixture indicates that the reaction mixture is liquid in the range between 40–220 °C, then confirming the eutectic mixture. In the 1:1 molar ratio, the broad peak may correspond to the small fraction of insolubilized SA in the eutectic mixture.

¹H-NMR analysis of upstream.

¹H-NMR analyses conducted in a Bruker Avance 400 MHz spectrometer. Aliquots of 20 µL of the reaction mixtures, SA and EtOH standards were dissolved in DMSO-d_6 up to 500 µL final volume using *tert*-butanol (100 mM) as internal standard. After a through mixing, the mixture was centrifuged at 15000 rpm, 5 °C, 5 min, to separate the solid TOPO. For TOPO analysis, chloroform-d_6 was selected as solvent. **Figure S3** describes a representative ¹H-spectrum containing SA and ester species.

¹H-NMR δ (ppm): EtOH: H_A 1.05 (t, 3H), H_B 3.44 (dq, 2H), Hc 4.36 (s, 1H). SA: H_A 2.39 (s, 4H). *tert*-butanol: H_A 1.10 (s, 9H), H_B 4.19 (s, 1H). Monoethyl succinate: H_A 1.17 (t, 3H), H_B 4.05 (q, 2H), H_C and H_D 2.46 (m, 4H, indistinguishables). Diethyl succinate: H_A 1.17 (t, 6H), H_B 4.05 (q, 4H), H_C 2.53 (s, 4H).

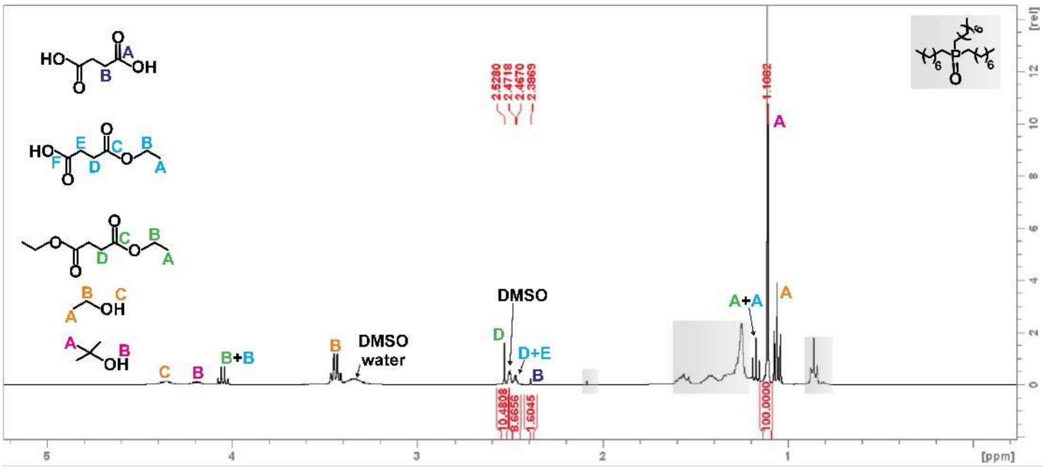

Figure S3. ¹H-NMR analysis. Representative ¹H-NMR spectrum of the reaction medium containing SA, ester species, EtOH and *tert*-butanol as internal standard, as well as the assignation of protons of the reaction species. TOPO signals are observed due to the presence of TOPO particles in suspension in the NMR tube.

Viscosity determinations

TOPO, 1:1 and 2:1 TOPO:SA (mol:mol) mixtures were selected to determine their viscosity using a Modular Compact Rheometer (MCR 102, Anton Paar) with a cone and plate geometry. A shear rate from 1 to 100 s^{-1} was used, collecting a total of 100 measurements for each sample within a range of 200 s at 60 °C (the reaction temperature).

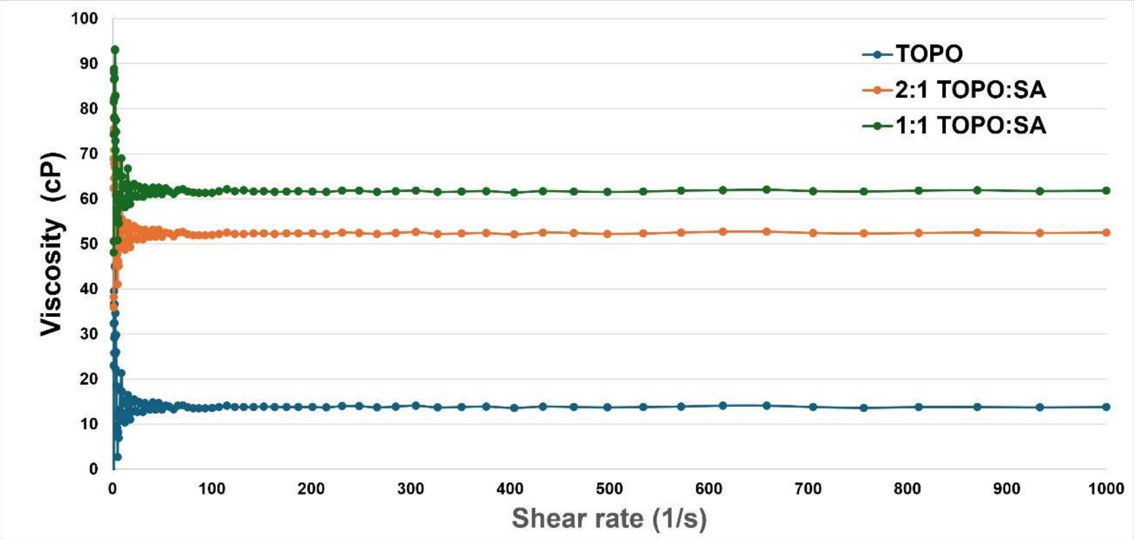

Figure S4. Viscosity of TOPO and eutectic mixtures. The linear relationship between the shear rate (1/s) and viscosity (cP) reveals a Newtonian behavior of the eutectic mixtures.

Influence of water addition as cosolvent

Two series of 1:1 and 2:1 TOPO: SA molar ratio were selected as representatives of partial and full eutectic mixtures, respectively. Water was added as cosolvent at different concentrations (respect the overall mass) to interrupt hydrogen interactions between TOPO and SA, determining water activity before and after the reaction. **Figure S5** shows the content of DEtS and MEtS obtained after the biocatalytic esterification.

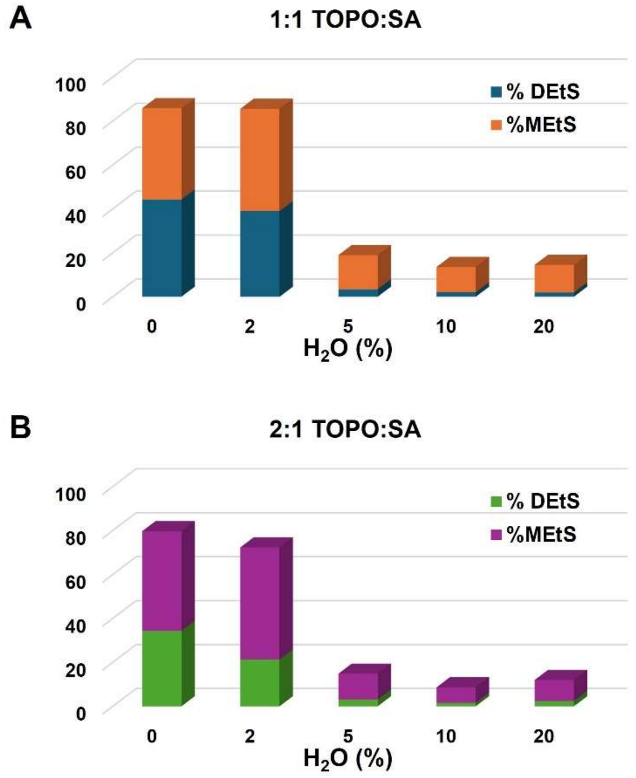

Figure S5. DEtS and MEtS synthesis in presence of water as cosolvent. (**A**). 1:1 TOPO: SA molar ratio with different amounts of added water. (**B**). 2:1 TOPO: SA molar ratio with different amounts of added water. The eutectic mixtures can stand up to 2% water. From 5% water content (0.8–0.9 a_w) both conversion and selectivity are dramatically affected although a 10–20% synthesis can yet be observed.

Green Metrics analyses

The analysis with Green Metrics was determined according to **Table S1**. The PMI value was calculated using the ACS PMI calculator (https://www.acs.org/content/dam/acsorg/greenchemistry/industriainnovation/roundtable/convergent-pmi-tool.xlsx).

Table S2. Green metric parameters equations [22,40].

Parameter	Calculation	
Atom Economy (AE)	$AE = \frac{Products\ Mw}{\Sigma \text{ Reactants Mw}}$	(S1)
Stoichiometric Factor (SF)	$SF = 1 + \frac{Mass\ of\ Excess\ of\ Reactives\ (g)}{Mass\ of\ Stoichiometric\ Reactives\ (g)}$	(S2)
Conversion (Conv)	$Conv = \frac{\text{mass of SA in products}}{\text{total mass of SA}}$	(S3)
Material Recovery Parameter (MRP)	$MRP = \frac{1}{1 + \frac{\varepsilon \cdot AE \cdot (C + S + W)}{SF \cdot MSP}}$	(S4)
Reaction Mass Efficiency (RME)	$RME = \frac{\varepsilon \cdot AE \cdot MRP}{SF}$	(S5)
E-factor (E-f)	$E = \frac{\text{mass of waste}}{\text{mass of products}}$	(S6)
Process Mass Intensity (PMI)	$PMI = E - factor + 1$	(S7)
Total Carbon Release (TCR)	$TCR = 2.3 \cdot PMI\ org + 0.63 \cdot PMI\ aq$	(S8)

Table S2 summarizes the calculated mass of the products, solvents, and waste streams of each reaction. N435 was included as waste because its reuse was not demonstrated yet, although this work has shown its stability in the rDES. Since TOPO was completely recovered, it was excluded from the calculations. It should be noted that no water-removal system was employed in either approach.

Table S3. Mass-balance of strategies selected for sustainability comparison.

		Waste				
**Entry**	**Products (g)**	**Water byproduct (g)**	**Solvent (g)**	**N435 (g)**	**Non-reacted SA (g)**	**Non-reacted Alcohol (g)**
**This work**	0.288 (DEtS) + 0.181 (MEtS)	0.08	-	0.099	0.028	0.626
**Abdul Rahman *et al.* [37]**	0.526	0.031	3.305	0.02	0.018	1.638
**Delhomme *et al.* [31]**	0.121	0.013	5	0.04	0.047	3.815

DEtS: Diethyl succinate; MEtS: Monoethyl succinate
